# Supplementary material for: Observation of perfect diamagnetism and interfacial effect on the electronic structures in infinite layer Nd0.8Sr0.2NiO2 superconductors
Source: Nat Commun. 2022 Feb 8;13:743. doi: 10.1038/s41467-022-28390-w (PMC8825820; doi:10.1038/s41467-022-28390-w)
Supplement: Supplementary file 1 — Supplementary Information [file 41467_2022_28390_MOESM1_ESM.pdf]

Supplementary Information

Observation of perfect diamagnetism and interfacial effect on the electronic structures in infinite layer $Nd_{0.8}Sr_{0.2}NiO_2$ superconductors

S. W. Zeng,[1, #,*] X. M. Yin,[2,3,#] C. J. Li,[4,5,#], L. E. Chow,[1,#] C. S. Tang,[2,6] K. Han,[1,7] Z. Huang,[1,7] Y. Cao,[8] D. Y. Wan,[1] Z. T. Zhang,[1] Z. S. Lim,[1] C. Z. Diao,[2] P. Yang,[2,4] A. T. S. Wee,[1,2] S. J. Pennycook[4], A. Ariando,[1,*]

[1]*Department of Physics, Faculty of Science, National University of Singapore, Singapore 117551, Singapore*

[2]*Singapore Synchrotron Light Source (SSLS), National University of Singapore, 5 Research Link, Singapore 117603, Singapore*

[3]*Shanghai Key Laboratory of High Temperature Superconductors, Physics Department, Shanghai University, Shanghai 200444, China*

[4]*Department of Materials Science and Engineering, National University of Singapore, Singapore 117575, Singapore*

[5]*Department of Materials Science and Engineering, Southern University of Science and Technology, Shenzhen 518055, Guangdong, China*

[6]*Institute of Materials Research and Engineering, A*STAR (Agency for Science, Technology and Research), 2 Fusionopolis Way, Singapore, 138634 Singapore*

[7]*Information Materials and Intelligent Sensing Laboratory of Anhui Province, Institutes of Physical Science and Information Technology, Anhui University, Hefei 230601, Anhui, China*

[8]*Department of Electrical and Computer Engineering, National University of Singapore, Singapore 117583, Singapore*

[#]The authors contributed equally to this work.

*To whom correspondence should be addressed: ariando@nus.edu.sg; phyzen@nus.edu.sg

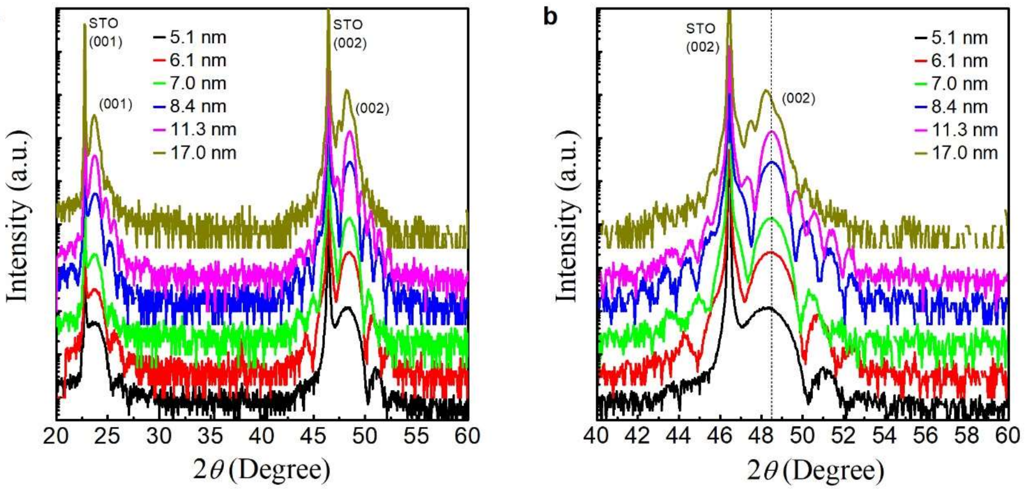

Supplementary Figure 1: (a) The XRD θ-2θ scan patterns of the as-grown $Nd_{0.8}Sr_{0.2}NiO_3$ thin films with different thicknesses on STO substrates. The intensity is vertically displaced for clarity. Only the (00l) perovskite peaks are observed, where l is an integer, confirming the c-axis oriented epitaxial growth. (b) The zoomed-in XRD θ–2θ scan patterns at angles from 40 to 60 degrees. The black dash line shows the position of the (002) perovskite peaks for thin films with different thicknesses. One can see that for thin films with thicknesses from 5.1 to 11.3 nm, the (002) peaks are at the same position, indicating the same c-lattice constant. For the film with a thickness of 17 nm, the presence of multiple peaks is seen at (002) position, suggesting the presence of mixed phases as the film thickness is increased, consistent with previous reports. The film thickness is calculated by fitting the Laue fringes in the vicinity of the (002) peak.

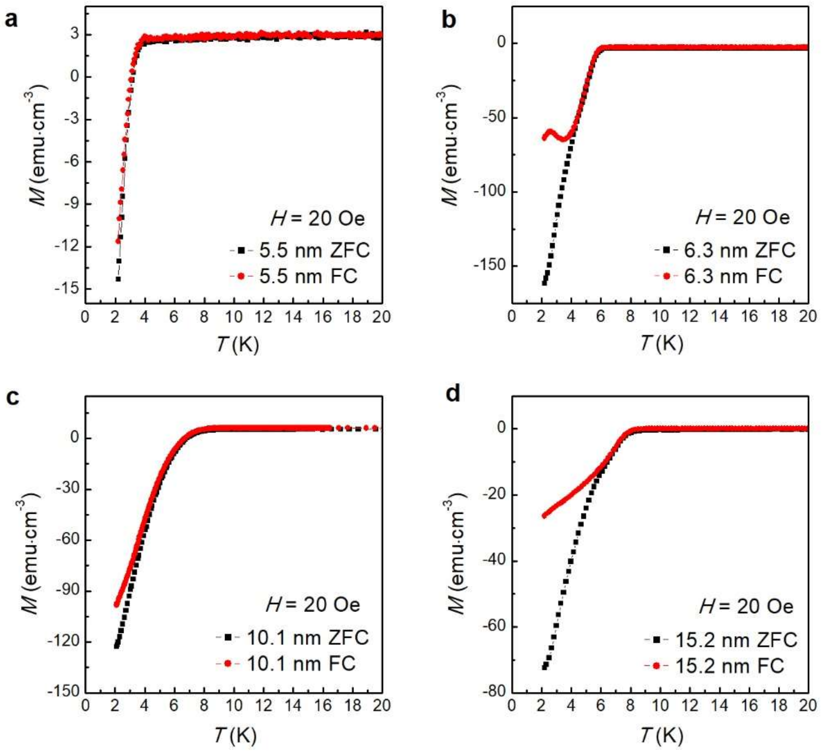

Supplementary Figure 2: Temperature dependence of magnetization (*M*-*T* curve) with zero-field cooling (ZFC) and field cooling (FC) for $Nd_{0.8}Sr_{0.2}NiO_2$ thin films with different thickness of (a) 5.5 nm, (b) 6.3 nm, (c) 10.1 nm and (d) 15.2 nm. The *M*-*T* curves are shown at temperatures from 20 to 2.1 K. The measurement and cooling fields are 20 Oe. The magnetic field is applied perpendicularly to the *a*-*b* plane.

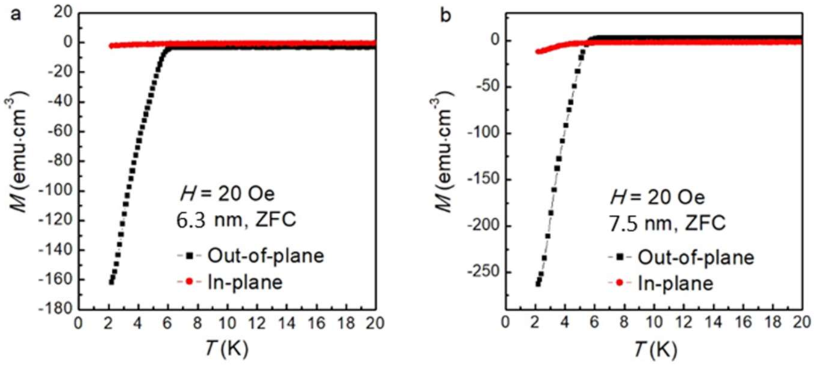

Supplementary Figure 3: Temperature dependence of magnetization with zero-field cooling (ZFC) mode for $Nd_{0.8}Sr_{0.2}NiO_2$ films with thickness of 6.3 nm and 7.5 nm. The magnetizations are shown for magnetic field perpendicular (out-of-plane) and parallel (in-plane) to the ab plane.

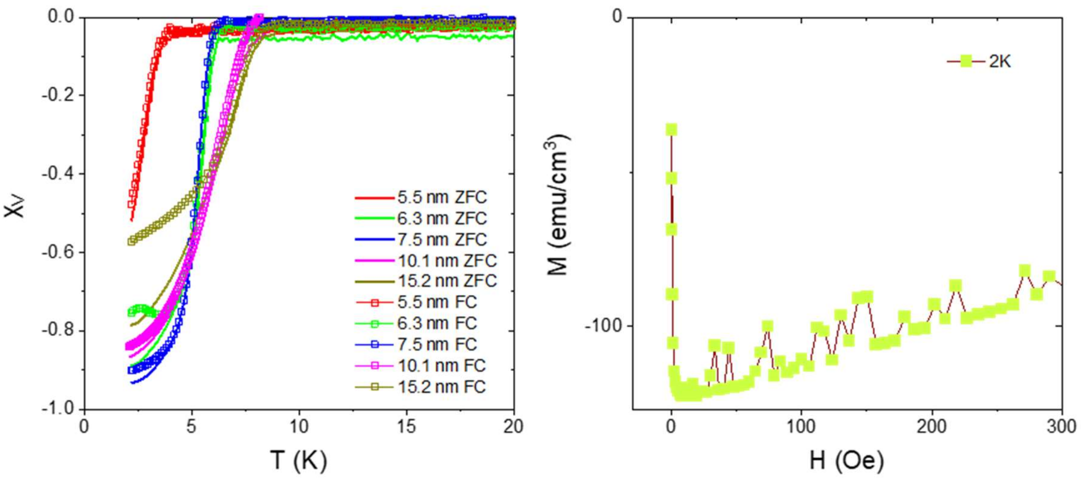

Supplementary Figure 4: (Left) Volume susceptibility as a function of temperature calculated from susceptibility measurement under $\mathrm{H} \mid\mid \mathrm{c} = 20\ \mathrm{Oe}$ in ZFC and FC conditions. In S.I. unit, $\chi_V = -1$ indicates 100% superconducting volume fraction (perfect diamagnetism). **(Right)** Magnetisation as a function of applied magnetic field at the out-of-plane direction in ZFC conditions. The STO substrate diamagnetic contribution is subtracted from the raw data with linear fitting of the gradient at large magnetic field range.

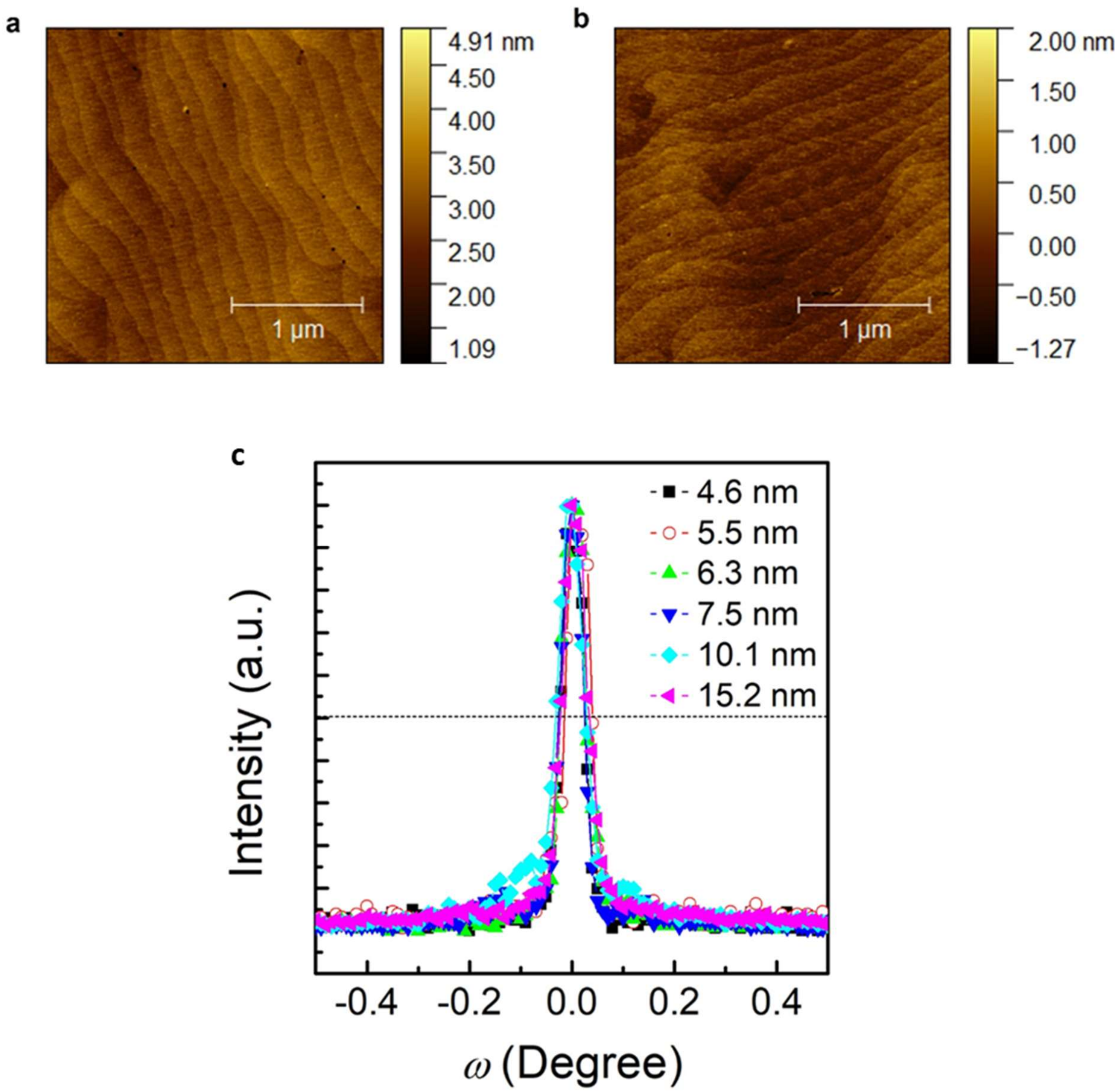

Supplementary Figure 5: (a-b) AFM images of the 6.3 nm sample surface (a) before (b) after reduction. **(c)** The full width at half-maximum (FWHM) of the (002) rocking curves. The value of FWHM is less than 0.06, indicating a good quality of the infinite-layer film.

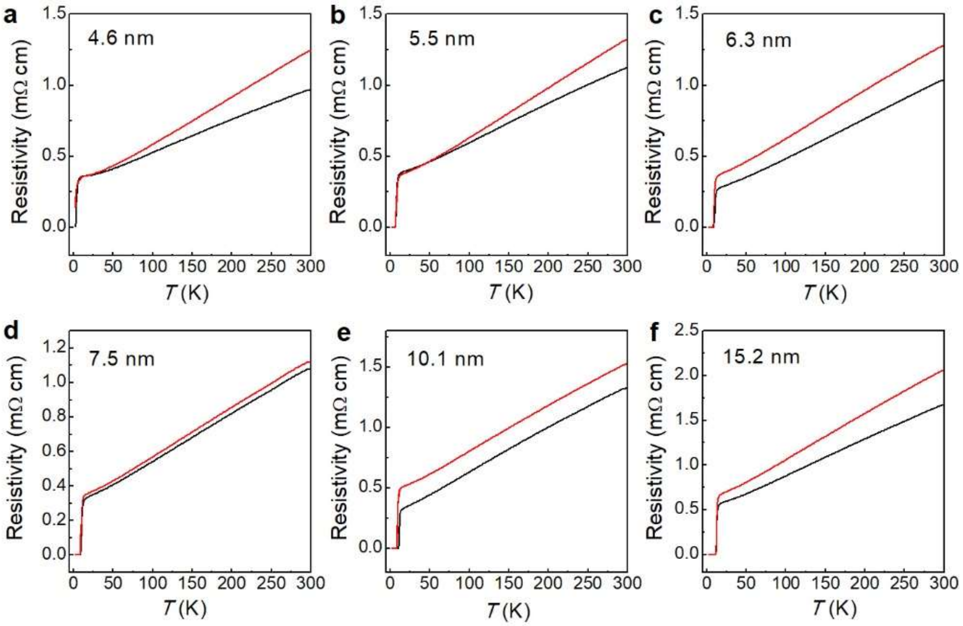

Supplementary Figure 6: The resistivity versus temperature (ρ-T) curves another set of samples. The black curves are the data shown in the main text, and the red curves are for another set of samples.
